# Preventive Effect of Molecular Iodine in Pancreatic Disorders from Hypothyroid Rabbits

**DOI:** 10.3390/ijms241914903

**Published:** 2023-10-05

**Authors:** Julia Rodríguez-Castelán, Evangelina Delgado-González, Esteban Rodríguez-Benítez, Francisco Castelán, Estela Cuevas-Romero, Brenda Anguiano, Michael C. Jeziorski, Carmen Aceves

**Affiliations:** 1 Instituto de Neurobiología, Universidad Nacional Autónoma de México, Juriquilla 76230, Querétaro, Mexico; julie.rocas@hotmail.com (J.R.-C.); edelgado@comunidad.unam.mx (E.D.-G.); anguianoo@unam.mx (B.A.); jeziorsk@unam.mx (M.C.J.); 2 Centro Tlaxcala de Biología de la Conducta, Universidad Autónoma de Tlaxcala, Tlaxcala 90070, Tlaxcala, Mexico; stivens.12emsad@gmail.com (E.R.-B.); fcocastelan@iibiomedicas.unam.mx (F.C.); ecuevas@uatx.mx (E.C.-R.); 3 Departamento de Biología Celular y Fisiología, Instituto de Investigaciones Biomédicas, Universidad Nacional Autónoma de México, Tlaxcala 90070, Tlaxcala, Mexico

**Keywords:** iodine, pancreatitis, hypothyroidism, rabbits

## Abstract

Pancreatic alterations such as inflammation and insulin resistance accompany hypothyroidism. Molecular iodine (I_2_) exerts antioxidant and differentiation actions in several tissues, and the pancreas is an iodine-uptake tissue. We analyzed the effect of two oral I_2_ doses on pancreatic disorders in a model of hypothyroidism for 30 days. Adult female rabbits were divided into the following groups: control, moderate oral dose of I_2_ (0.2 mg/kg, M-I_2_), high oral dose of I_2_ (2.0 mg/kg, H-I_2_), oral dose of methimazole (MMI; 10 mg/kg), MMI + M-I_2,_, and MMI + H-I_2_. Moderate or high I_2_ supplementation did not modify circulating metabolites or pancreatic morphology. The MMI group showed reductions of circulating thyroxine (T4) and triiodothyronine (T3), moderate glucose increments, and significant increases in cholesterol and low-density lipoproteins. Acinar fibrosis, high insulin content, lipoperoxidation, and overexpression of GLUT4 were observed in the pancreas of this group. M-I_2_ supplementation normalized the T4 and cholesterol, but T3 remained low. Pancreatic alterations were prevented, and nuclear factor erythroid-2-related factor-2 (Nrf2), antioxidant enzymes, and peroxisome proliferator-activated receptor gamma (PPARG) maintained their basal values. In MMI + H-I_2,_ hypothyroidism was avoided, but pancreatic alterations and low PPARG expression remained. In conclusion, M-I_2_ supplementation reestablishes thyronine synthesis and diminishes pancreatic alterations, possibly related to Nrf2 and PPARG activation.

## 1. Introduction

Preclinical and clinical studies have described a close relationship between thyroid status and pancreatic function [1,2]. Thyroid hormones are critical for the normal development and function of the endocrine and exocrine pancreas during the neonatal period [3]. In adults, thyroid hormones induce pancreatic acinar cell proliferation [4]. Hypothyroidism is accompanied by pancreatic alterations such as pancreatitis, insulitis, insulin resistance, and type 2 diabetes mellitus (T2DM) [1,5,6]. Pharmacological hypothyroidism modifies insulin secretion and the expression of GLUT4, hexokinase, and glucokinase [6,7,8,9,10]. Previous studies have reported that methimazole-induced hypothyroidism in female rabbits promotes interstitial edema and degenerative changes in pancreatic acinar cells [11,12]. Many of these alterations could be explained by the direct effect of thyroid hormones on differentiation target genes and their impact on the oxidative state. In the thyroid and other organs, the inflammation (edema, vascularization, and infiltration) observed during hypothyroidism has been explained by the low expression of antioxidant thyroid-dependent enzymes such as catalase (Cat) and glutathione peroxidase [8].

Moreover, some authors have postulated that the pancreatic redox equilibrium could be modulated by the local presence of iodine per se. Iodine may have an ancestral antioxidant function in all iodide-concentrating cells, from primitive algae to more recent vertebrates [13]. In the chemical form of molecular iodine (I_2_), its reductive capacity in vitro (by the ferric reducing antioxidant power assay) is ten times more efficient than ascorbic acid and 100 times more potent than potassium iodide, while in vivo, it binds reactive oxygen species, thereby neutralizing °OH radicals, resulting in less cellular oxidative damage [14]. It has also been demonstrated that I_2_ acts as a direct activator of the nuclear factor erythroid-2-related factor-2 (Nrf2) pathway, triggering the expression of several phase II protective antioxidant enzymes such as superoxide dismutase type 1 (Sod1) and Cat [15]. In addition, I_2_ can bind to arachidonic acid and activate the peroxisome proliferator-activated receptor type gamma (PPARG), prompting metabolic, antioxidant, and immuno-regulatory effects [16]. Studies have only recently begun analyzing the mechanisms involved in the actions of iodine in the pancreas. Thus, in the spontaneous type 1 diabetes model (Bio-Breeding/Worcester rats, or BB rats), moderate iodine supplementation prevented the incidence of this pathology, decreasing insulitis [17]. Similar results were described in the murine model of streptozotocin-induced pancreatitis, where inflammation processes and fibrosis activation by stellar pancreatic cells were prevented [18]. Moreover, it is well established that B-cells exhibit a continuous expression of deiodinase type 3 (Dio3), which, in addition to regulating the active thyroid hormones, generates local concentrations of free iodine [19]. The purpose of the present study was to evaluate oral I_2_ supplementation in pancreatic disorders associated with hypothyroidism.

## 2. Results

**Figure 1:**
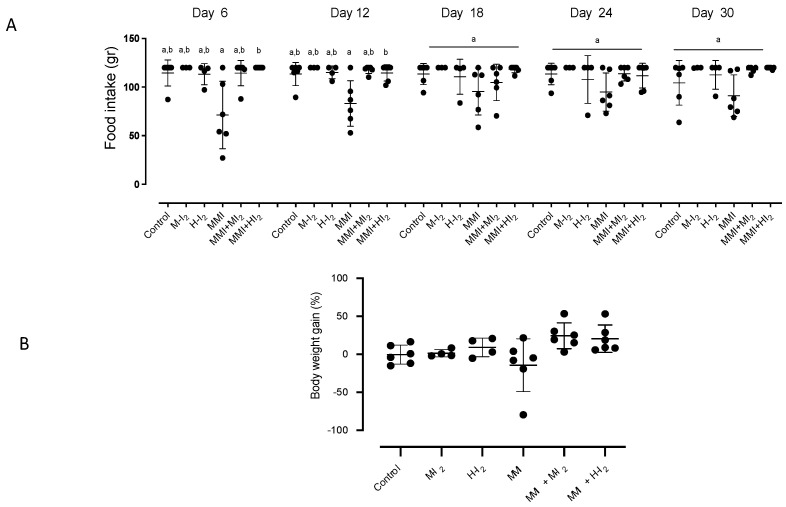
Food consumed and body weight gain for all groups. Control, moderate oral dose of I_2_ (M-I_2_, 0.2 mg/kg); high oral dose of I_2_ (H-I_2_, 2.0 mg/kg); oral dose of methimazole (MMI, 10 mg/kg); MMI + M-I_2_; and MMI + H-I_2_. (**A**) The changes in food intake during the entire treatment. (**B**) Final body weight gain on sacrifice day. Results are expressed as mean ± SD. Different letters denote statistical differences (one-way ANOVA and Kruskal–Wallis; *p* < 0.05).

Figure 1 shows the food consumption and body weight gain for all groups. During the first two weeks, the MMI treatment resulted in reduced food intake, which could be attributed to the sour taste of MMI. However, no significant changes were observed compared to the control group (Figure 1A). The body weight gain was similar between groups (Figure 1B).

Table 1 summarizes the serum parameters. Serum alterations did not result from I_2_ supplementation at moderate or high concentrations. In contrast, the decrease in thyroid hormone values confirmed the hypothyroid state of animals undergoing the MMI treatment in comparison with control animals (T3: 60.9 ± 2.3 vs. 79.6 ± 4.1 and T4: 1.4 ± 0.2 vs. 2.7 ± 0.5, respectively). Moreover, the increase in TC (105.4 ± 5.5 vs. 74.3 ± 0.8) and LDL-C (35.2 ± 3.6 vs. 12.4 ± 1.5) values is indicative of dyslipidemia and the significant increase in serum soluble CD163 protein (0.40 ± 0.08 vs. 0.04 ± 0.1) could be associated with macrophage activation and general inflammatory status. The coadministration of MMI with a moderate dose of I_2_ (MMI + M-I_2_) prevented the decline in T4 (2.5 ± 0.3) and kept the dyslipidemia and inflammation markers at basal values (TC: 63.5 ± 7.0 and LDL-C: 10.5 ± 1.9; sCD163: 0.07 ± 0.01), but without fully reestablishing T3 values (59.7 ± 3.2). The high I_2_ supplement with MMI (MMI + H-I_2_) maintained the serum concentration of both thyroid hormones (T3: 75.8 ± 5.3 and T4: 2.3 ± 0.5), TC (75.8 ± 5.3) and LDL-C (7.8 ± 0.5) at basal values, suggesting that I_2_ supplementation at this concentration avoids the establishment of hypothyroidism. Moreover, the decrease in TAG (29.3 ± 8.2 vs. 72.5 ± 4.9) and VLDL (8.2 ± 1.0 vs. 14.5 + 1.1), as well as the increase in the HDL-C (55.8 ± 2.9 vs. 46.3 ± 3.9), suggest a modulation in hepatic lipid metabolism generated by iodine concentrations per se. Although the serum glucose concentration did not show statistical differences, a moderate increase in the MMI group was normalized with the iodine supplements.

**Figure 2:**
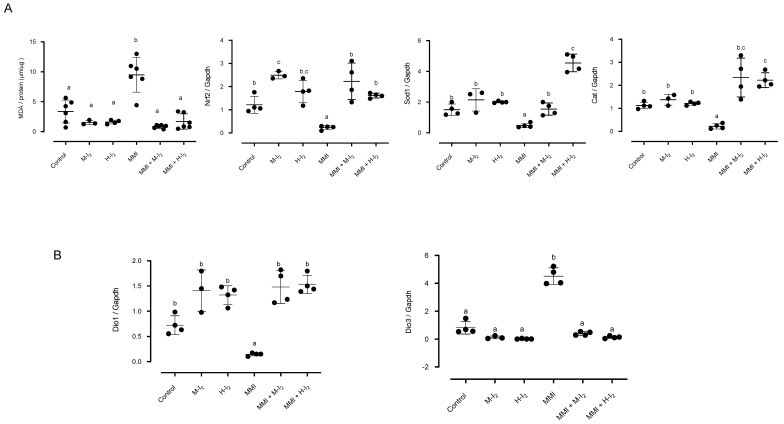
Pancreatic status in antioxidants and deiodinases for all groups. Control, moderate oral dose of I_2_ (M-I_2_, 0.2 mg/kg); high oral dose of I_2_ (H-I_2_, 2.0 mg/kg); oral dose of methimazole (MMI, 10 mg/kg); MMI + M-I_2_; and MMI + H-I_2_. (**A**) Lipoperoxidation [expressed as micromoles of malondialdehyde (MDA) per micrograms of protein], NF-E2-related factor 2 (Nrf2), superoxide dismutase (Sod1), and catalase (Cat) expression (qRT-PCR). (**B**) deiodinase type 1 (Dio1) and type 3 (Dio3) expression (qRT-PCR). Results are expressed as mean ± SD. Different letters denote statistical differences (One-way ANOVA and Kruskal-Wallis; *p* < 0.05).

Pancreas tissue analysis (Figure 2A) showed that M-I_2_ and H-I_2_ groups did not exhibit significant differences in oxidative status compared to control animals. In contrast, the MMI group showed the highest lipoperoxidation (MDA: 10.5 ± 1.7 vs. 4.5 ± 0.5) and the lowest values of Nrf2 (0.2 ± 0.08 vs. 1.2 ± 0.3), Sod1 (0.06 ± 0.02 vs. 1.3 ± 0.4), and Cat (0.2 ± 0.07 vs. 1.1 ± 0.01), corroborating the oxidative status that is characteristic of the hypothyroid condition. Local hypothyroidism is consistent with the differential expression of pancreatic deiodinases: low Dio1 (0.3 ± 0.07 vs. 1.4 ± 0.2) and high Dio3 (2.4 ± 0.7 vs. 0.8 ± 0.2) (Figure 2B).

The moderate I_2_ dose showed that although circulating T3 does not rise to basal values, the normalized T4 may be enough to prevent the decrease in Dio1 (1.4 ± 0.2) and the increases in Dio3 (1.0 + 0.08)—additionally, maintaining the pancreatic lipoperoxidation at basal values (MDA; 0.85 ± 0.11) and the normal expression of Nrf2 (2.2 + 0.7), Sod1 (1.8 + 0.5), and Cat (2.3 ± 0.8). The high I_2_ supplement, which was accompanied by basal circulating levels of thyroid hormones, exerted preventive effects on lipoperoxidation (1.3 ± 0.5), Dio1 (0.8± 0.1), and Dio3 (1.2 ± 0.09) values, and elicited increases in Nrf2 (1.6 ± 0.1) and Cat (2.2 ± 0.3), showing the most elevated values in Sod1 (4.3± 0.7) and suggesting a pancreatic euthyroid and activated redox status.

Table 2 shows the analysis of pancreatic islets. The study involved measuring 100–250 islets per group. Moderate or high I_2_ doses did not impact the percentage of islets, regardless of their size or the mean length of each category. However, MMI-induced hypothyroidism (MMI group) caused a reduction in the number of cells in small islets, which suggests decreased cell proliferation. The moderate I_2_ dose prevented the reduction, whereas the high dose did not.

Since the moderate or high dose of I_2_ did not modify the circulating or pancreatic parameters, our subsequent analysis focused on these groups: Control, MMI, MM + M-I_2_, and MMI + H-I_2_.

**Figure 3:**
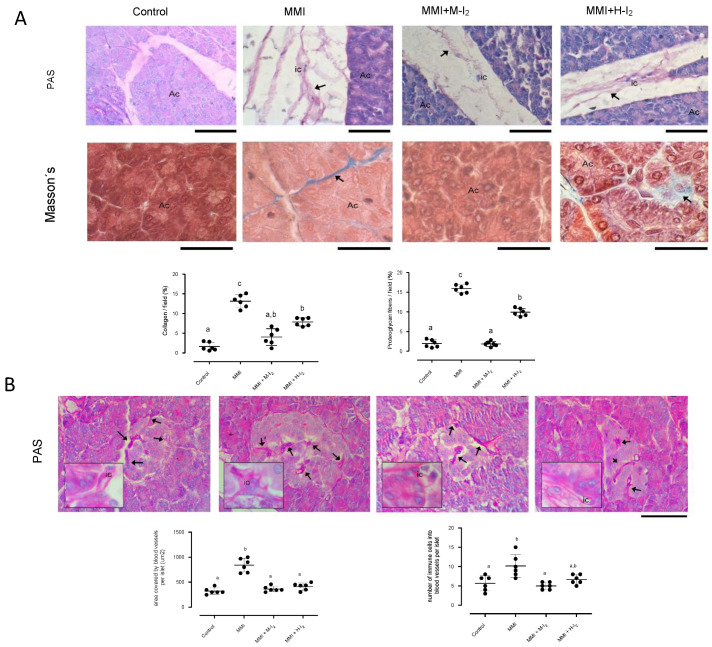
Impact of I_2_ treatment on inflammation indicators in the pancreas. Control, moderate oral dose of I_2_ (M-I_2_, 0.2 mg/kg); high oral dose of I_2_ (H-I_2_, 2.0 mg/kg); oral dose of methimazole (MMI, 10 mg/kg); MMI+ M-I_2_; and MMI+ M-H_2_. (**A**) Pancreatic acinus microphotographs show proteoglycans (black arrows in PAS stain) and collagen (black arrows in Masson’s trichrome). Scale: PAS stain 50 µm; Masson stain 20 µm. Abbreviations: acinar cells (Ac), immune cells (ic). Quantitative analysis (graphics) of proteoglycans and collagen proportion (%) was performed as the average of three random regions (40×) for each animal using the ImageJ 1.47 program. (**B**) PAS stain of pancreatic islets microphotographs showing blood vessels (black arrows) and immune cell infiltration (ic). Quantitative analysis (graphics) corresponds to the area covered by blood vessels into islets and the infiltration of immune cells inside blood vessels. Results are expressed as mean ± SD. Different letters denote statistical differences (one-way ANOVA, Tukey’s test; *p* < 0.05).

Figure 3 shows the status of the pancreatic acinus. Compared with the control group, the MMI group increased the area covered by collagen deposits (14.8 + 0.05 vs. 2.4 + 0.2) and proteoglycan fibers (17.3 + 0.06 vs. 2.4 + 0.03) on interlobular septa (Figure 3A). Moreover, a significant increase was observed in the area covered by blood vessels (823 ± 38 vs. 292 ± 41) and in the number of immune cells in the blood vessels of islets (9.3 ± 1.5 vs. 4.6 ± 0.3; Figure 2B) in the MMI group compared with the control group. Moderate I_2_ supplementation reduced collagen and proteoglycan deposits to basal levels, while high I_2_ supplementation could not recover the acinar injury induced by MMI. However, this high I_2_ dose maintained the basal amount of blood vessels and immune cell infiltration in islets.

**Figure 4:**
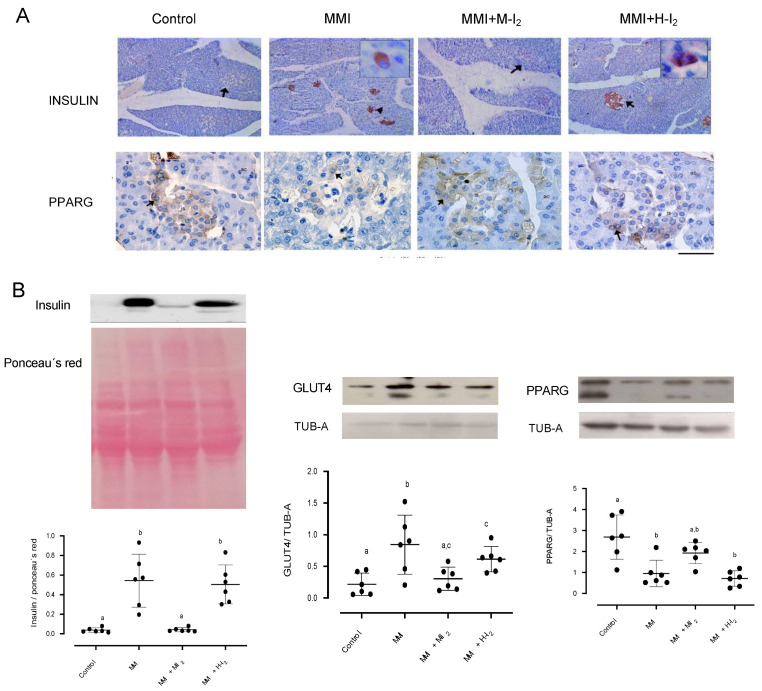
Impact of I_2_ treatment on insulin, GLUT4, and PPARG expressions in the pancreas. Control, moderate oral dose of I_2_ (M-I_2_, 0.2 mg/kg); high oral dose of I_2_ (H-I_2_, 2.0 mg/kg); oral dose of methimazole (MMI, 10 mg/kg); MMI+ M-I_2_; and MMI+ H-I_2_. (**A**) representative immunohistochemistry for insulin and PPARG protein (positive cells; black arrows) Scale: 50 µm. (**B**) Western blotting for insulin, GLUT4, and PPARG in the whole pancreas. Values were normalized using alpha-tubulin (TUB-A) as an internal control. Results are expressed as mean ± SD. Different letters denote statistical differences (one-way ANOVA, Tukey’s test; *p* < 0.05).

The image in Figure 4 depicts the immunoreactivity of insulin and PPARG and the expression of insulin, PPARG, and GLUT4 proteins in whole pancreas tissue. The MMI group had a higher expression of insulin (0.56 + 0.09 vs. 0.06 + 0.02) and GLUT4 (0.95 + 0.09 vs. 0.21 + 0.06) than the control group, indicating a possible alteration in insulin synthesis and delivery. This group also exhibited a decreased PPARG expression (1.0 + 0.02 vs. 2.8 + 0.05). Moderate I_2_ concentrations prevented the increase in insulin and GLUT4 expression caused by hypothyroidism and retained the PPARG amount (2.1 + 0.07). However, the high I_2_ dose did not prevent the rise in insulin (5.2 + 0.07) and GLUT4 (0.6 + 0.02) or the reduction of PPARG expression (0.9 + 0.05) caused by MMI treatment.

## 3. Discussion

The present study confirms that hypothyroidism injures pancreatic physiology and that the oxidative stress observed in this condition could be related to altered signaling in the Nrf2/Keap1/ARE pathway with deficient expression of antioxidant enzymes such as Sod1 and Cat, as has been previously suggested [7,8]. The evident attenuation of some pancreatic damage observed in animals supplemented with moderate doses of I_2_ could be partly explained by the re-establishment of synthesis and circulating levels of thyroid hormone. These data are consistent with previous work in which we demonstrated that iodide transporters such as NaI-symporter (NIS) or Pendrin do not uptake I_2_, but the thyroid gland can uptake this form of iodine through a facilitating mechanism [20]. This agrees with data found in a family with a specific inactivated NIS mutation, but the consumption of Laminaria algae, which contain different chemical forms of iodine (including I_2_), attenuated the hypothyroidism syndrome associated with this alteration [21]. Our results indicate that this oxidized chemical form of iodine does not require thyroid peroxidase, which is inhibited by MMI, to bind thyroglobulin-generating T4 and T3. The present work also shows that moderate I_2_ supplementation partially reestablished euthyroid pancreatic status by preventing the decrease in Dio1 and maintaining the high expression of Sod1 and Cat; however, it could not normalize Dio3. Nevertheless, the moderate dose of I_2_ was adequate to prevent circulating lipid alterations (cholesterol and TAG). Indeed, the normalized values of sCD163, a biomarker of macrophage activation in various inflammatory diseases (e.g., macrophage activation syndrome and sepsis), and during the development of T2DM [22,23] are consistent with the prevention observed in almost all pancreatic damage such as fibrosis, collagen, and immune infiltrations. In addition, these normalized values restored pancreatic functionality by decreasing insulin storage and GLUT4 overexpression. This glucose transporter has been related to the energy required for glucagon and insulin synthesis [24].

Although we cannot separate the effect of thyroid hormones reestablished from the moderated I_2_ dose, several reports have described the direct actions of iodine per se. Hypercholesterolemia was reduced in overweight women with iodine supplementation [25]. Moderate iodine diets improve the lipid profile in mice, increasing LDLR and scavenger receptor class B type-1 in the liver [26]. In addition, part of the effects observed with moderate I_2_ supplementation could be explained by the activation of PPARG receptors. I_2_ supplementation is accompanied by 6-iodolactone (6-IL) formation in the mammary gland. 6-IL is an iodolipid derived from arachidonic acid and an agonist ligand of PPARG [27]. It has been proposed that the improvement in glucose homeostasis observed with thiazolidinediones, PPARG agonists, could be related to enhanced B-cell function. The activation of PPARG in B-cells involves the induction of anti-inflammatory mechanisms [28], the reduction of oxidative stress [29], and the inhibition of amyloid formation [30].

Moreover, the prevention of inflammation and fibrosis in the pancreatic acini observed in the MMI + M-I_2_ group could be associated with the anti-inflammatory actions of I_2_. In vitro, I_2_ promotes the release of anti-inflammatory cytokines such as interferon-gamma, interleukin 6 (IL6), IL10, and IL8-CXCL8 in normal lymphocytes [31]. The protection against fibrosis formation could also be explained by the activation of PPARG by stimulating phosphatase and tensin homolog expression, which decreases the TGFB1 and PI3K/Akt pathways [32]. It has been described that pancreatic stellate cell (PSc) activation promotes the failure of B-cell function and increases fibrosis [33]. Natural compounds with antioxidant properties, such as resveratrol and curcumin, can inhibit the activation of these cells and diminish the production of reactive oxygen species and collagen in vitro [34,35] and in mice with cerulein-induced chronic pancreatitis [36].

Hypothyroidism increases the expression of GLUT4 and insulin. Considering that glucose is the most crucial factor in regulating the architecture of islets, the moderate dose of I_2_ restored the effects found in the MMI group. It has been described that B-cell mass and islets increase during the first stage of T2DM. This increase is accompanied by greater insulin production [37,38] or excessive synthesis of misfolded proinsulin [39]. A new formation of islets has been observed in the hypothyroid pancreas [10]. Two mechanisms are involved in new islet formation: 1) the replication of preexisting B-cells and neogenesis [40] or 2) A-cell phenotype modification to B-cells through the activation of Arx and Dnmt1 genes to regenerate islet function [41]. In this sense, in our previous studies using the murine model of streptozotocin-induced pancreatitis, I_2_ supplementation prevented the increase in the number of A-cells, thereby decreasing the inflammation and canceling the fibrosis activation by PSc [18]. We also found that I_2_ modulates the cell cycle and participates in the transdifferentiation of the cell population through PPARG activation in cancerous mammary cells [16]. Lipotoxicity has been proposed as a mechanism of B-cell failure, mainly through ceramides and oxidative lipid production, promoting alterations in the mitochondria and nucleus [42,43]. In this regard, I_2_ supplementation inhibits lipid peroxidation in both normal and cancer cells [14,18], and we observed the same effect in the present work with both I_2_ concentrations, indicating a sustained antioxidant effect.

On the other hand, the combination of MMI and high doses of I_2_ maintained a euthyroid state, but it was accompanied by differential serum and pancreatic alterations that were not observed with I_2_ alone (H-I_2_ group). The MMI + H-I_2_ group showed almost all circulating parameters in the normal range, including elevated HDL-C and lower triglycerides and VLDL-C, indicating a favorable lipid metabolism. However, the high concentration of I_2_ was unable to prevent the increased amount of collagen and proteoglycans in the interlobular septa of pancreatic tissue, as well as the infiltration of immune cells, even with the increased response on antioxidant signaling (Nrf2, Sod1, and Cat), suggesting that these pancreatic damages could be related to the extrathyroidal effects of MMI + H-I_2_. Several recent reports have indicated that MMI treatment could be accompanied by pancreatic injury, and one was administered with elevated concentrations of iodine [44]. The mechanism involved in this combination is unknown, but the inhibition of PPARG expression could explain this unexpected result. Studies that directly correlate MMI exposure with the inhibition of PPARG actions do not exist, but it has been described that low PPARG expression increases PSc proliferation and activation, generating high fibrosis and collagen deposits [42].

These results show that I_2_ supplementation at moderate doses prevents some metabolic and pancreatic alterations associated with hypothyroidism. The anti-inflammatory and lipid modulation effects could be related to antioxidant Nrf2 mechanisms and PPARG activation. The therapeutic effect of I_2_ in chronic pancreatic diseases related to inflammation is currently being analyzed.

## 4. Materials and Methods

### 4.1. Animals

Adult chinchilla-breed virgin female rabbits (*Oryctolagus cuniculus*) of 8–9 months were housed under controlled temperature (20 ± 2 °C) conditions and a 16:8 h light: dark cycle. Animals were provided pellet food (120 g/day) and water ad libitum. Rabbits were randomly assigned to the following experimental groups: control (control; *n* = 6), moderate oral (drinking water) dose of I_2_ (0.2 mg/kg; M-I_2_; *n* = 3), a high oral dose of I_2_ (2.0 mg/kg; H-I_2_; *n* = 4); oral dose of methimazole (10 mg/kg; MMI; *n* = 6), MMI + M-I_2_ (*n* = 6), and MMI + H-I_2_ (*n* = 6). The body weight of the females was measured before and at the end of treatments. After four weeks of treatment, the rabbits were anesthetized with sodium pentobarbital (90 mg/kg, i.p.) and euthanized with an overdose of the same anesthetic. The Ethics Committee at Universidad Autónoma de Tlaxcala approved this experimental design following the guidelines of Mexican Law for the Production, Care, and Use of Laboratory Animals. Immediately after death, the left lobe of the pancreas was collected, histologically processed, embedded in Paraplast X-TRA (Sigma-Aldrich, St Louis, MO, USA), and longitudinally cut at a thickness of 5 μm using a microtome (Thermo Scientific, Model Finesse 325, Waltham, MA, USA). The right lobe of the pancreas was frozen at −80 °C for biochemical measures.

### 4.2. Thyroid Hormones and Metabolic and Inflammatory Variables

Blood samples were obtained by cardiac puncture from rabbits fasted for 12 h at the end of the experiment. The serum concentration of total thyroxine (T4) and total triiodothyronine (T3) were measured using ELISA (International Immuno-Diagnostics, Foster City, CA, USA). Glucose, total cholesterol (TC), and triacylglycerol (TAG) were measured using standard enzymatic methods (ELITech, Puteaux, France). High-density lipoprotein cholesterol (HDL-C) was measured by a precipitating method (ELITech, Puteaux, France). The concentrations of low and very low-density lipoproteins (LDL-C and VLDL-C) were calculated from the concentration of TAG using the Friedewald equations: [VLDL-C] = 0.2 × [TAG], and [LDL-C] = [TC] − [HDL-C] − [VLDL] [8]. Serum soluble CD163 protein was measured by Western blot using goat antibody (1:200 dilution, sc-18794, Santa Cruz Biotechnology, Dallas, TX, USA) and donkey anti-goat antibody-HRP (1:3000 dilution; sc-2020, Santa Cruz Biotechnology). The protein preparation was performed according to the Western blot method described below and normalized by ~90% of the total protein content (Ponceau).

### 4.3. Expression of Deiodinases and Antioxidant Enzymes

Pancreas tissue (~50 mg) was homogenized in TRIzol reagent according to the manufacturer’s protocol to extract RNA (Life Technologies, Inc., Carlsbad, CA, USA). RNA quantity and purity were measured using a NanoDrop 2000 spectrophotometer (Thermo Scientific, Waltham, MA, USA). Two micrograms of RNA were used to synthesize first-strand cDNA with the Moloney murine leukemia virus (M-MLV) reverse transcriptase (Invitrogen, Carlsbad, CA, USA). Amplification of Nrf2, Dio1, Dio3, Sod1, and Cat was carried out with the primers described in Table 3. As the internal control, the expression level was normalized using glyceraldehyde 3-phosphate dehydrogenase (Gapdh) amplification. The Rotor-Gene 3000 apparatus (Corbett Research, Mortlake, NSW, Australia) was employed to perform quantitative real-time PCR with a marker for DNA amplification (SYBR Green, Fermentas, Burlington, ON, Canada) [18].

### 4.4. Protein Extraction and Western Blotting

Pancreas tissue (~50 mg) was disrupted using an electronic homogenizer (TissueTearor, BioSpec Products, Inc., Bartlesville, OK, USA) in lysis buffer (20 mM Tris-HCl pH 7.4, 100 mM glycine, 100 mM NaCl, 0.1% triton X-100, 1 mM phenylmethylsulfonyl fluoride, and 1 mM DL-dithiothreitol) supplemented with protease inhibitor cocktail. For insulin measurement, 30 or 80 µg of protein extracts were denatured in Laemmli sample buffer, resolved in 10% or 16% SDS-PAGE plus 6 M urea, and electro-blotted onto nitrocellulose membranes (Bio-Rad Laboratories, Hercules, CA, USA). Membranes were stained with Ponceau’s Red to confirm that all samples had equal protein content. Membranes were soaked with 3.0% non-fat dry milk plus 2.0% bovine serum albumin or 17% non-fat dry milk, all diluted in phosphate-buffered saline containing 0.2% tween-20 (PBST). Then they were incubated overnight at 4 °C as indicated: anti-insulin (1:200 dilution, ab6995, Abcam), anti-GLUT4 (1:100 dilution, ab48547, Abcam, Cambridge, UK), and anti-PPARG (1:1000 dilution, sc-7196, Santa Cruz Biotechnology). Later, the membrane was incubated with a secondary antibody (1:5000 dilution, sc-2005 goat anti-mouse, or 1:20000 dilution, sc-2004 goat anti-rabbit, Santa Cruz Biotechnology) conjugated with horseradish peroxidase at room temperature under constant agitation for 1 h. Immunoreactivity was enhanced by chemiluminescent substrate (West Pico Signal, Thermo Scientific, Rockford, IL, USA), and images were captured and analyzed with a transilluminator and software (MyECL Imager, Thermo Fisher Scientific; Waltham, CA, USA). Insulin was normalized against the signal obtained from the Ponceau Red staining used as a loaded control [12]. To correct differences in the total protein loaded in each lane, protein content for GLUT4 and PPARG was normalized using alfa tubulin (TUB-A) as an internal control. Blots were stripped with a 0.1 M glycine solution (pH 2.5, 0.5% SDS) for two hours at 37 °C and incubated with anti-TUB-A (1:1000 dilution, sc-5286, Santa Cruz Biotechnology) at 4 °C. Blots were incubated with a secondary antibody (1:5000 dilution, sc-2005 goat anti-mouse) at room temperature for one hour under constant agitation. 

### 4.5. Immunohistochemistry

Slides of pancreas samples were deparaffinized and incubated in microwave-heated 10 mM sodium citrate pH 6 to retrieve antigens. Endogenous peroxidases were quenched with 0.3% hydrogen peroxide diluted in PBS. Endogenous binding sites for secondary antibodies were blocked with 5% normal donkey serum or normal goat serum diluted in PBS with 0.3% Triton X-100 (PSBT). Independent sections were incubated with anti-insulin (1:50 dilution, ab6995, Abcam) and anti-PPARG (1:200) diluted in PBST for 16 h at 4 °C. Subsequently, they were incubated with secondary antibodies (1:250 goat anti-mouse for Insulin; and 1:2000 goat anti-rabbit for PPARG) diluted in PBST for 2 h at 37 °C. Immunostaining was developed according to the Vectastain ABC kit instructions (Vector Labs, Burlingame, CA, USA) using 0.05% 1,3′-diaminobenzidine (Sigma-Aldrich) and 0.01% H_2_O_2_ as enzyme substrate. Sections were counterstained with Mayer’s hematoxylin. Non-specific immunostaining was observed when primary antibodies were omitted. Sections were observed under a light microscope (Zeiss Axio Imager A1, Oberkochen, Germany) and photographed using a digital camera (Jenoptik, Jena, Germany) at 10×, 40×, or 100×.

### 4.6. Acinar and Islet Morphology

Morphometric analysis of islets was performed using an optical microscope at 4× (Zeiss Axio Imager A1). Pancreas samples were stained with either Masson’s trichrome to identify the presence of collagen or Periodic Acid-Schiff (PAS) to analyze proteoglycans and quantify the proportion (%) from three random regions (40×) from each animal using the ImageJ 1.47 program. Samples stained with PAS were counterstained with Mayer’s hematoxylin to evaluate the morphometry of islets, blood vessels, and proteoglycans. Images from each pancreas were reconstructed. A random selection of these reconstructions permitted us to measure the cross-sectional area and count the number of cells for different islets in photographs at 40×. Islets were classified as small (<4000 µm^2^), medium (4000–7000 µm^2^), and large (>7000 µm^2^) [11]). Moreover, in three selected areas per animal, photographed at 40×, we evaluated the area covered by intra-acinar collagen and proteoglycan and blood vessels and immune cells in islets [12].

### 4.7. Lipids and Peroxidation in the Pancreas

The pancreas sample (25 mg) was homogenized in ice-cold Tris buffer (20 mM, pH 7.4) and centrifuged at 3000 rpm for 10 min at 4 °C. The supernatant was collected and immediately tested with the lipid peroxidation microplate assay (Oxford Medical Research, Inc., San Louis, MO, USA). The kit uses the thiobarbituric acid reaction, and lipoperoxidation is expressed as micromoles of malondialdehyde (MDA) per microgram of protein of the pancreas.

### 4.8. Statistical Analyses

Statistical analyses were performed with GraphPad Prism v6.01 (GraphPad Software, La Jolla, CA, USA). The Kolmogorov–Smirnov test was used to check the normality of distribution. Results were expressed as mean ± SD. One-way ANOVA and Tukey’s post hoc test, or Kruskal–Wallis’s test, were used to determine significant differences between groups (*p* < 0.05). 

## 5. Conclusions

These results show that I_2_ supplementation at moderate doses prevents some metabolic and pancreatic alterations associated with hypothyroidism. The anti-inflammatory and lipid modulation effects could be related to antioxidant Nrf2 mechanisms and PPARG activation.

## Figures and Tables

**Table 1:** Serum concentrations of thyroid hormones and metabolic variables from all groups.

Variable	Control	M-I_2_	H-I_2_	MMI	MMI + M-I_2_	MMI + H-I_2_
T3 (ng/dL)	79.6 + 4.1 ^a^	75.2 + 7.6 ^a^	74.5 + 4.8 ^a^	60.9 + 2.3 ^b^	73.0 + 3.9 ^a^	75.8 + 5.3 ^ab^
T4 (ug/dL)	2.7 + 0.5 ^a^	2.4 + 0.4 ^a^	2.5 + 0.2 ^a^	1.4 + 0.2 ^b^	2.5 + 0.3 ^a^	2.3 + 0.5 ^a^
Glucose (mg/dL)	113.2 + 5.1 ^a^	122 + 32 ^a^	119 + 23.6 ^a^	138.2 + 4.5 ^a^	113.0 + 5.9 ^a^	114.3 + 1.7 ^a^
Total cholesterol (mg/dL)	74.3 + 0.8 ^a^	67.6 + 7.2 ^a^	70.4 + 12.7 ^a^	105.4 + 5.5 ^b^	63.5 + 7.0 ^a^	75.9 + 2.7 ^a^
Triacylglycerol (mg/dL)	72.5 + 4.9 ^a^	70.8 + 16.2 ^a^	50.9 + 8.7 ^a^	94.5 + 3.1 ^c^	36.8 + 2.6 ^b^	29.3 + 8.2 ^b^
LDL-C (mg/dL)	12.4 + 1.5 ^a^	11.7 + 1.7 ^a^	13.2 + 3.9 ^a^	35.2 + 3.6 ^b^	10.5 + 1.9 ^a^	7.8 + 0.5 ^a^
HDL-C (mg/dL)	46.3 + 3.9 ^a^	42.1 + 7.5 ^a^	34.3 + 5.9 ^a^	44.9 + 4.1 ^a^	58.9 + 2.0 ^b^	55.8 + 2.9 ^b^
VLDL-C (mg/dL)	14.5 + 1.1 ^a^	14.2 + 3.2 ^a^	10.2 + 1.7 ^a^	13.8 + 1.7 ^a^	10.5 + 1.2 ^a^	8.2 + 1.0 ^a^
sCD163	0.04 + 0.01 ^a^	0.04 + 0.01	0.05 + 0.01	0.40 + 0.08 ^b^	0.07 + 0.01 ^a^	0.14 + 0.03 ^a^

Data are mean ± SD (*n* = 3–6). Different letters indicate statistically significant differences (*p* < 0.05). LDL-C, low-density lipoprotein cholesterol; HDL-C, high-density lipoprotein cholesterol; VLDL-C, very-low-density lipoprotein cholesterol; SCD163, soluble CD163 protein; M-I_2_, moderated oral dose of I_2_ (0.2 mg/kg); H-I_2_, high oral dose of I_2_ (2.0 mg/kg); MMI, oral dose of methimazole (10 mg/kg).

**Table 2:** Pancreatic islet analysis.

Morphometric Variable	Control	M-I_2_	H-I_2_	MMI	MMI + M-I_2_	MMI + H-I_2_	Statistics
Cross-sectional area (CSA) of islets (µm^2^)	6218 ± 715 ^a^	7756 ± 130 ^a^	7495 ± 233 ^a^	5752 ± 860 ^a^	6922 ± 878 ^a^	7173 ± 1182 ^a^	K = 3.5; *p* = 0.62
% Mean of small islets <4000 µm^2^	44.9 ± 6.6 ^a^	6.8 ± 0.5 ^b^	14.4 ± 2.2 ^ab^	52.7 ± 1.8 ^a^	25.6 ± 5.5 ^ab^	35.4 ± 10.1 ^ab^	K = 19.2; *p* = 0.001
% Mean of medium islets 4000–7000 µm^2^	22.5 ± 3.3 ^a^	17.6 ± 3.0 ^a^	21.8 ± 5.4 ^a^	21.4 ± 1.2 ^a^	36.5 ± 4.5 ^a^	22.8 ± 5.0 ^a^	K = 8.0; *p* = 0.15
% Mean of large islets >7000 µm^2^	32.6 ± 6.9 ^ab^	75.5 ± 2.7 ^a^	63.9 ± 5.6 ^ab^	25.9 ± 2.9 ^b^	37.9 ± 6.3 ^ab^	41.8 ± 9.9 ^ab^	K = 14.5; *p* = 0.01
Mean CSA (µm^2^) for small islets	2478 ± 221 ^ab^	2350 ± 74 ^ab^	3011 ± 126 ^a^	1971 ± 131 ^b^	2645 ± 272 ^ab^	2328± 118 ^ab^	K = 12.1; *p* = 0.03
Mean CSA (µm^2^) for medium islets	5524 ± 257 ^a^	5624 ± 86 ^a^	5445 ± 138 ^a^	5437 ± 88 ^a^	5579 ± 147 ^a^	5503 ± 271 ^a^	K = 1.5; *p* = 0.90
Mean CSA (µm^2^) for large islets	11,046 ± 827 ^a^	14,696 ± 349 ^a^	14,029 ± 657 ^a^	16,603 ± 2194 ^a^	9977 ± 1226 ^a^	11,909 ± 58 ^a^	K = 13.6; *p* = 0.01
Mean number of cells in small islets	24.3 ± 1.7 ^a^	19.2 ± 3.3 ^ab^	22.6 ± 1.5 ^ab^	16.6 ± 0.9 ^b^	21.4 ± 2.5 ^ab^	17.1 ± 1.3 ^ab^	K = 16.1; *p* = 0.01 *
Mean number of cells in medium islets	45.4 ± 3.7 ^a^	33.2 ± 1.0 ^a^	36.9 ± 3.5 ^a^	45.2 ± 5.3 ^a^	43.4 ± 2.4 ^a^	40.8 ± 4.7 ^a^	K = 6.0; *p* = 0.29
Mean number of cells in large islets	103.1 ± 5.3 ^a^	75.6 ± 13.8 ^a^	93.9 ± 10.5 ^a^	84.9 ± 5.1 ^a^	83.8 ± 9.6 ^a^	75.7 ± 8.2 ^a^	K = 7.3; *p* = 0.19

Different letters and *, denote statistical differences; Dunn’s post-hoc test.

**Table 3:** Primers used for gene amplifications (qRT-PCR).

Gen	Reference	Primer Sequence	bp
Nrf2	XM_008258785.3	FW: 5′-TGTGCTGTCAAGGGACATGG-3′ RV: 5′-GTGTTGGGCTGGCTGAATTG-3′	241
Cat	XM_002709045.4	FW: 5′- AGAGTCGCTGCATCAGGTTT-3′ RV: 5′-CGTCCAAGAGGGGTAGTTGC-3′	265
Sod1	NM_001082627.2	FW: 5′-CTTCGAGCAGAAGGGAACAGG-3′ RV: 5′-GCTGCCTGCAGTCACATTAC-3′	214
Dio1	NM_001099958.1	FW: 5′-GCCAGAAGACCGGGATAGC-3′ RV: 5′-GGTGCTGAAGAAGGTGGGAAT-3′	71
Dio3	XM_008248683.3	FW: 5′-TCTACATTGAGGAGGCGCAC-3′ RV: 5′-ACATGATGGTGCCACTCTGG-3′	225
Gapdh	NM_001082253.1	FW: 5′-TCGGAGTGAACGGATTTGGC-3′ RV: 5′- CCAGCATCACCCCACTTGAT-3′	256

Nrf2, nuclear factor erythroid-2-related factor-2; Cat, catalase; Sod1, superoxide dismutase 1; Dio1, deiodinase 1; Dio3, deiodinase 3; Gapdh, glyceraldehyde 3-phosphate dehydrogenase; bp, base pair.

## Data Availability

The data presented in this study are available on request from the corresponding author.
